# Selective Deposition of SiO_2_ on Ion Conductive Area of Soda-lime Glass Surface

**DOI:** 10.1038/srep27767

**Published:** 2016-06-13

**Authors:** Daisuke Sakai, Kenji Harada, Yuichiro Hara, Hiroshi Ikeda, Shiro Funatsu, Keiichiro Uraji, Toshio Suzuki, Yuichi Yamamoto, Kiyoshi Yamamoto, Naoki Ikutame, Keiga Kawaguchi, Hideo Kaiju, Junji Nishii

**Affiliations:** 1 Department of Electrical and Electronic Engineering, Kitami Institute of Technology, 165 Koen-cho, Kitami, Hokkaido 090-8507, Japan; 2 Department of Computer Science, Kitami Institute of Technology, 165 Koen-cho, Kitami, Hokkaido 090-8507, Japan; 3 Art, Science and Technology Center for Cooperative Research, Kyushu University, 6-1 Kasuga-koen, Kasuga, Fukuoka 816-8580, Japan; 4 Innovative Technology Research Center, Asahi Glass Co., Ltd., 1-1 Suehiro-cho, Tsurumi-ku, Yokohama, Kanagawa, 230-0045, Japan; 5 Innovative Technology Research Center, Asahi Glass Co., Ltd., 1150 Hazawa-cho, Kanagawa-ku, Yokohama, Kanagawa, 221-8755, Japan; 6 Research Institute for Electronic Science, Hokkaido University, Kita20, Nishi10, Kitaku, Sapporo, Hokkaido 001-0020, Japan

## Abstract

Selective deposition of SiO_2_ nanoparticles was demonstrated on a soda-lime glass surface with a periodic sodium deficient pattern formed using the electrical nanoimprint. Positively charged SiO_2_ particles generated using corona discharge in a cyclic siloxane vapor, were selectively deposited depending on the sodium pattern. For such phenomena to occur, the sodium ion migration to the cathode side was indispensable to the electrical charge compensation on the glass surface. Therefore, the deposition proceeded preferentially outside the alkali-deficient area. Periodic SiO_2_ structures with 424 nm and 180 nm heights were obtained using one-dimensional (6 μm period) and two-dimensional (500 nm period) imprinted patterns.

Fabrication of fine structures on oxide glass surfaces is attracting great attention for highly functional and reliable optics. Recently, thermal nanoimprinting has become used for the fabrication of fine structures on several oxide glasses^1^,^2^,^3^,^4^. However, the mold lifetime is insufficient because of the high process temperature. Xerography-like deposition has also been used for the periodical arrangement of nanoparticles^5^,^6^,^7^. Particles immersed in a solution can be patterned on the locally charged surface of the substrate. Electrospray deposition has also been studied for micropattern formation^8^,^9^,^10^. Despite benefits such as high throughput and wide-area patterning, these processes are disadvantageous for fabricating structures with a high aspect ratio, such as those required for microwavelength and sub-microwavelength optics, such as high spatial frequency gratings^11^, phase control plates^12^, anti-reflective lenses^13^,^14^, and strong plasmon coupled gratings^15^.

As described in this report, we propose a selective deposition method of SiO_2_ using a corona discharge for electrically imprinted soda-lime glass. The corona discharge is generated at the anode electrode with high voltage^16^,^17^,^18^,^19^, where the generated charged ion or particles are accelerated to the cathode electrode.

The electrical imprint can form fine structures on alkali-containing glasses at lower temperatures than those for thermal nanoimprinting^20^,^21^. The origin of structure formation is the diffusion of alkali ions in the glass from the anode side to the cathode side, depending on the mold pattern, which is similar to alkali ion behavior that occurs during thermal polling processing^22^,^23^,^24^,^25^. Using these two processes, we fabricated fine structures on soda-lime glasses at low temperatures and ambient pressures.

## Results

### Selective deposition

**Figure 1:**
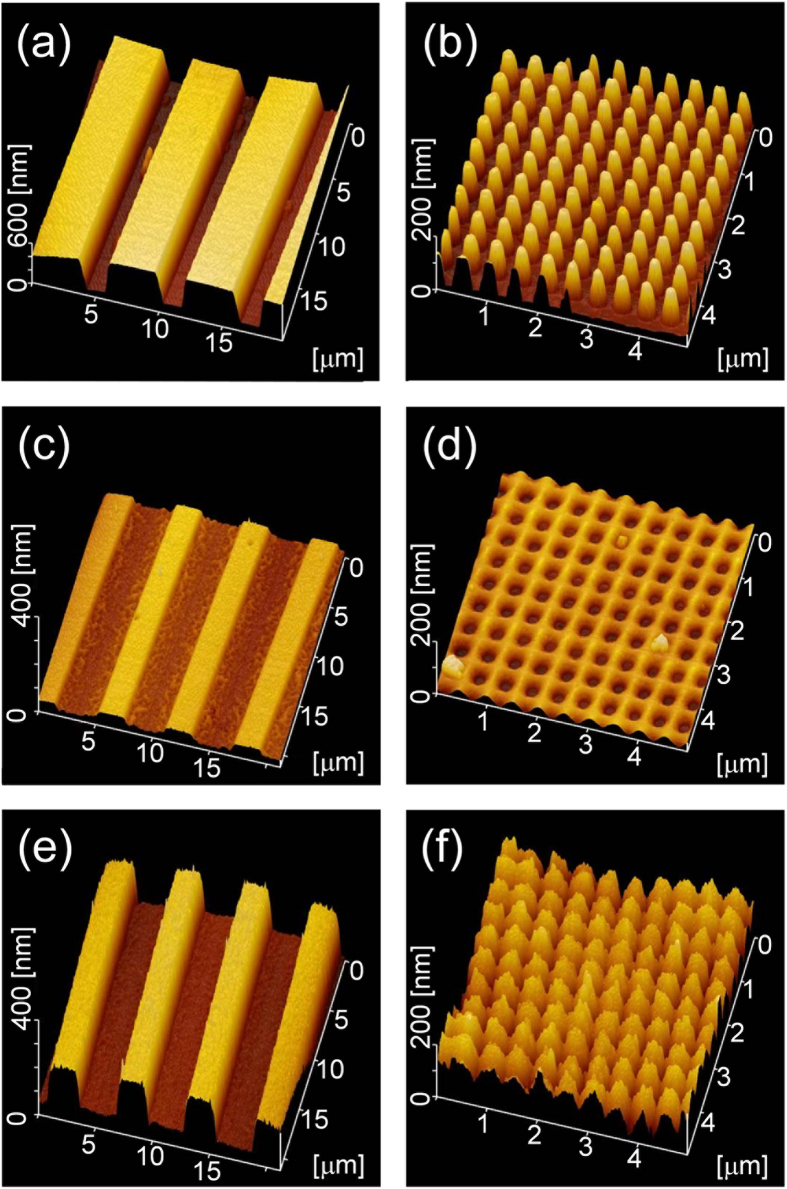
AFM views of molds (upper), glass surfaces after electrical imprint applying +200 V (middle), and after corona discharge in air containing cyclic siloxane vapor (lower): (**a**) mold A, one-dimensional with a 6 μm period; (**b**) mold B, two-dimensional with a 500 nm period; (**c**,**d**) respectively show the imprinted surfaces using molds A and B; (**e**,**f**) respectively show after corona discharge using imprint substrate (**c**,**d**).

Figure 1(c,d) show atomic force microscope (AFM, Nanocute; SII Nanotechnology Inc.) views of the surface profiles on the specimens electrically imprinted with the molds of two types: mold A, one-dimensional with a 6 μm period and mold B, two-dimensional with a 500 nm period as shown in Fig. 1(a,b). Shallow concavities were formed on the glass surface after the electrical imprint applying +200 V. The formation mechanism of concavities was reported in our previous paper^21^. Figure 1(e,f) present surface profiles after the corona discharge treatment by an application of +6 kV to the needle electrode for 60 min at 200 °C in air containing vaporized cyclic siloxane. The structure height on substrates imprinted under +200 V increased efficiently after the corona discharge without deviation of periods. The maximum heights were 175 nm for the one-dimensional pattern and 157 nm for the two-dimensional pattern.

**Figure 2:**
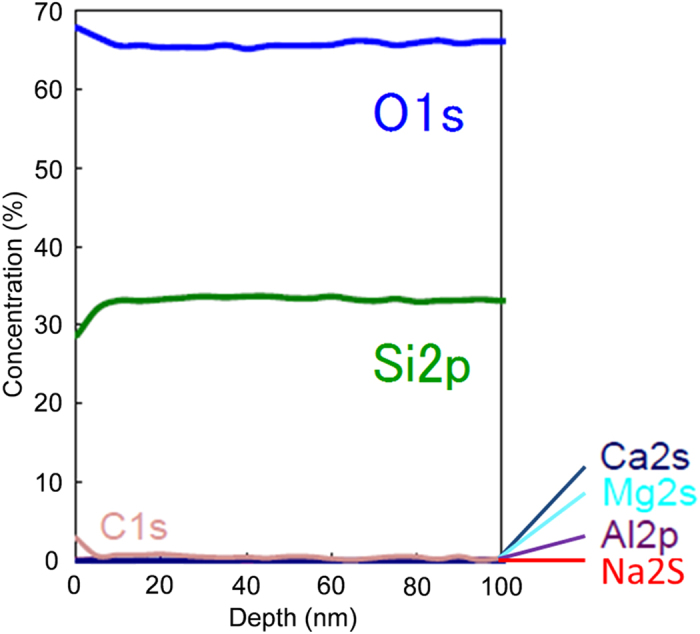
Compositional depth profile of material deposited using an XPS combined with C_60_ ion sputtering.

Chemical composition of the deposited area after the corona discharge treatment was analyzed using an X-ray photoelectron spectrometer (XPS) combined with C_60_ ion sputtering, as shown in Fig. 2^26^. Although a trace amount of carbon contamination from ambient was detected on the top surface, the main material was SiO_2_. In contrast, the SiO_2_ deposition on the glass was never recognized during the corona discharge treatment in an ambient air. Therefore, these results suggest that the vaporized cyclic siloxane was decomposed by the corona discharge plasma, and that it was deposited on the glass surface as SiO_2_.

### Mechanism of selective deposition

**Figure 3:**
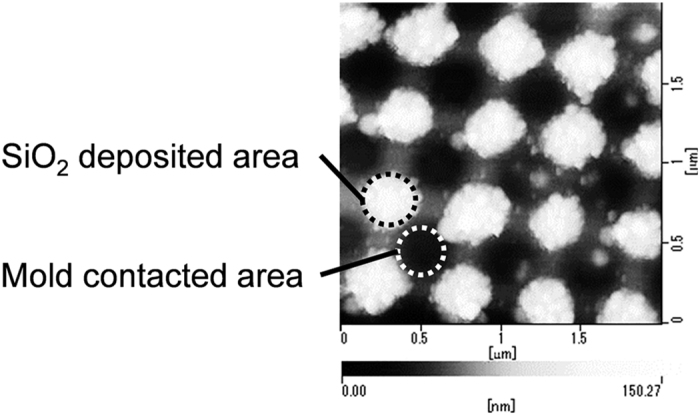
AFM view of the glass surface after the electrical imprinting applying +200 V using the mold B, followed by the corona discharge in air containing cyclic siloxane vapor for 30 min at 200 °C.

The next topic of interest is the selective deposition mechanism of SiO_2_ only on the electrically imprinted glass surface after the application of a positive voltage. Figure 3 presents an enlarged AFM view of the glass surface after the electrical imprint with +200 V using mold B (see Fig. 1(b)) followed by the corona discharge in cyclic siloxane vapor for 30 min at 200 °C. Deposition occurred clearly on the outside of the applied voltage area.

**Figure 4:**
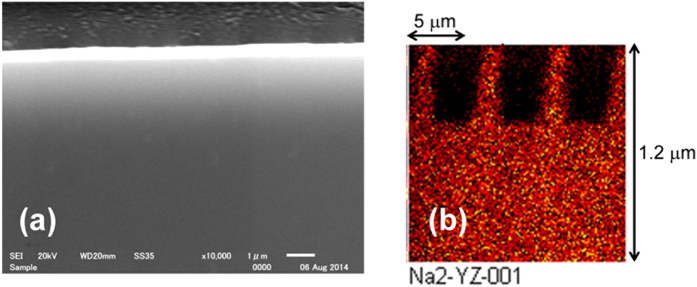
Cross-sectional images of electrically imprinted soda-lime glass: (**a**) surface profile observed using a SEM and (**b**) sodium ion distribution analyzed using a ToF-SIMS with C_60_ ion sputtering.

Stemmer *et al*.^5^ and Okuyama *et al*.^9^ respectively reported the selective deposition of silica nanoparticles on a substrate with a patterned surface charge using xerography-like deposition and electrospray deposition. They attributed the origin of selective deposition to the electrostatic attractive force between charges localized on a substrate and on the particles. In order to confirm the origin of the selective deposition, we investigated the surface charge on the electrically imprinted glass using a Kelvin force microscope (KFM, Nanocute; SII Nanotechnology Inc.). However, the measurement noise of KFM was larger than the expected surface charge. Furthermore, the selective chemical vapor deposition could not be confirmed on the glass after electrical imprinting with negative voltage. Therefore, no relation is expected to exist between the surface charge and the selective chemical vapor deposition. Instead, a plausible origin of the selective corona discharge deposition is the formation of pattern with different ion conductivity by the electrical imprint. Then the cross-sectional image of the glass was observed after the electrical imprint with +200 V using mold A. Figure 4(a) portrays the surface profile observed using a scanning electron microscope (SEM, JSM-6510A, JEOL Ltd.). It was hard to recognize the periodic concavo-convex structure on the surface. However, as shown in Fig. 4(b), there was a periodic sodium deficient pattern of 400 nm deep remained in the area contacted with the mold, which was analyzed using a time-of-flight secondary ion mass spectrometer (ToF-SIMS, TOF.SIMS5; ION-TOF GmbH Inc.) with C_60_ ion sputtering^27^.

**Figure 5:**
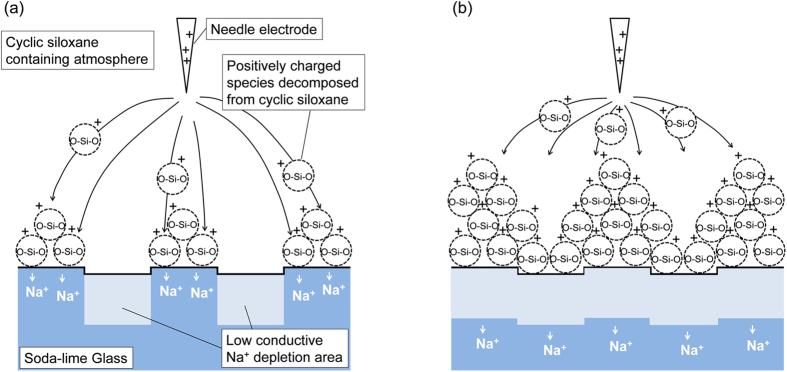
Schematic drawing of selective deposition of positively charged species decomposed from cyclic siloxane on soda-lime glass (**a**) in the early stage of selective deposition and (**b**) after prolonged corona discharge treatment.

Enami *et al*. reported that the conductivity of sodium depletion area was decreased by five orders of magnitude (from 10^−10^ to 10^−15^ S) at 180 °C compared with that of the pristine glass, which means that there is a pattern of ion conductivity depending on the sodium concentration^24^. On the other hand, the corona discharge plasma decomposes the cyclic siloxane vapor to a positively charged species, which is accelerated in the cathode direction and which is deposited selectively on the ion conductive area, i.e., a sodium ion area. The importance of the ion conductivity for the glass substrate can be demonstrated by the following experiment using two glasses with different conductivities. Namely, during the corona discharge treatment, the half area of soda-lime glass was covered with a dehydrated synthetic fused silica glass plate with a negligibly low ion conductivity. In consequence, the SiO_2_ deposited only on the soda-lime glass surface. Details were described in the Supplementary Information. Based on the considerations presented above, the selective deposition of SiO_2_ can be described as portrayed in Fig. 5(a).

### Deposition characteristics

**Figure 6:**
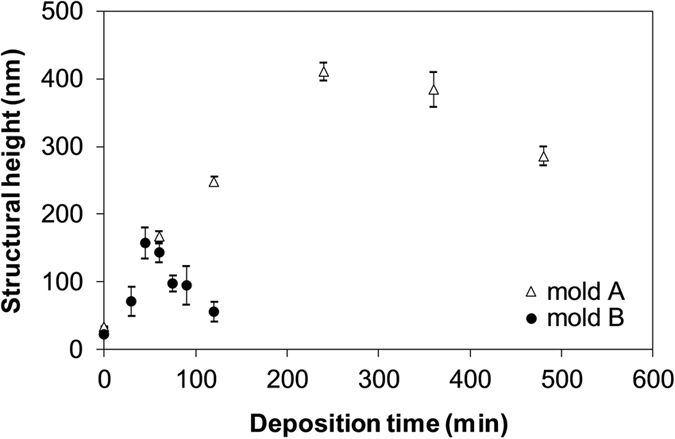
Relation between the selective deposition time and the structural height using mold A, one-dimensional with a 6 μm period, and mold B, two-dimensional with a 500 nm period.

Figure 6 shows the relation between the selective deposition time and the structural height when the corona discharge condition was +6 kV applied voltage at 200 °C. The maximum heights increased up to 424 nm and 180 nm for molds A and B, respectively. The heights decreased with the deposition time after their peak times. The selectivity of the deposition degraded with the deposition time gradually, and the grooves were filled by the SiO_2_ after the prolonged corona discharge treatment. The degradation of selective deposition should be caused by the equalization of surface ion conductivity because of the migration of remaining sodium ions in the electrically imprinted soda-lime glass to the cathode side as shown in Fig. 5(b). Therefore, the optimization of electrical imprint and corona discharge conditions, deposition species and substrate composition might realize the rapid formation of fine structures with a high aspect ratio.

## Conclusions

We proposed the fabrication process for fine structures using corona discharge selective chemical vapor deposition of SiO_2_ onto an electrically imprinted soda-lime glass surface. Sodium in the surface area of the glass contacted with the mold migrated to the cathode side using the electrical imprint. The vaporized cyclic siloxane was decomposed to positively charged silicon oxide by the corona discharge plasma, which were deposited selectively as SiO_2_ onto the sodium ion area on the soda-lime glass because of the high ion conductivity. The maximum structure heights were 424 nm and 180 nm for a one-dimensional mold with a 6 μm period and a two-dimensional mold with a 500 nm period, respectively. These SiO_2_ structures can be formed at 200 °C under the ambient pressure. A contrast of the ion conductivity in the glass determines the aspect ratio of the selective deposition structures. Furthermore, the optimization of the deposition condition might include factors such as the chemical composition of the glass, the vaporization source, and the state of ionization. Their optimization is expected to enable the production of the desired fine SiO_2_ structures for use in highly functional and reliable next-generation optics.

## Methods

**Figure 7:**
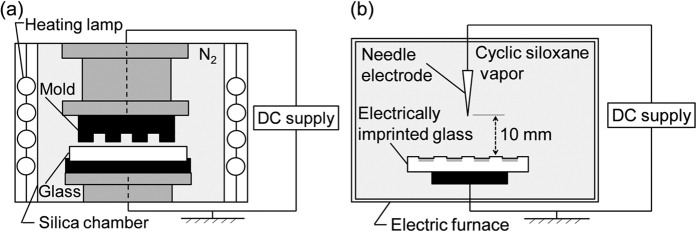
Schematic drawing of the experimental setup for (**a**) electrical imprint and (**b**) corona discharge treatment.

Commercially available soda-lime glass (Tg = 555 °C) of 10 mm × 10 mm × 1 mm was used for the substrate. The electrical imprint setup is presented in Fig. 7(a). In this work, SiO_2_ molds of two types were prepared using conventional photolithography and dry etching. The mold surface was coated with 40-nm-thick carbon by sputtering to maintain its electrical conductivity. The mold was then set in the electrical imprint machine and was connected with the DC power supply (Series EH; Glassman High Voltage Inc.). The glass sample fixed on the grounded stage was imprinted by the carbon-coated-mold at 3 MPa in pressing load, 450 °C for 180 s in N_2_ atmosphere. Then +200 V was applied to the mold during imprinting.

The electrically imprinted glass was subjected to corona discharge treatment in air containing cyclic siloxane vapor. Figure 7(b) shows the fundamental setup for the corona discharge treatment. A nickel-coated steel needle and an aluminum plate were set, respectively, in the electric furnace (FO200; Yamato Scientific Co. Ltd.) as anode and cathode electrodes. These electrodes were connected to a DC power supply (AKTB-010K1P; Towa Keisoku Co. Ltd.). The electrically imprinted glass sample was placed on the cathode electrode. The distance between the anode tip and the glass top surface was fixed at 10 mm. An adhesive of heat-resistant tape (Nitoflon Adhesive Tape No. 903UL, volatilization of adhesive: >180 °C; Nitto Denko Corp.) was used as the source of cyclic siloxane vapor in the chamber. The corona discharge was generated by an application of +6 kV at 200 °C in air containing cyclic siloxane vapor.

## Additional Information

**How to cite this article**: Sakai, D. *et al*. Selective Deposition of SiO_2_ on Ion Conductive Area of Soda-lime Glass Surface. *Sci. Rep.* **6**, 27767; doi: 10.1038/srep27767 (2016).

## Supplementary Material

Supplementary Information
