# Supplementary material for: Selective Deposition of SiO2 on Ion Conductive Area of Soda-lime Glass Surface
Source: Sci Rep. 2016 Jun 13;6:27767. doi: 10.1038/srep27767 (PMC4904222; doi:10.1038/srep27767)
Supplement: Supplementary Information [file srep27767-s1.pdf]

Supplementary Information

for Selective Deposition of SiO_2 on Ion Conductive Area of Soda-lime Glass Surface

Daisuke Sakai[1,*], Kenji Harada[2], Yuichiro Hara[2], Hiroshi Ikeda[3], Shiro Funatsu[4], Keiichiro Uraji[4], Toshio Suzuki[5], Yuichi Yamamoto[5], Kiyoshi Yamamoto[5], Naoki Ikutame[6], Keiga Kawaguchi[6], Hideo Kaiju[6], and Junji Nishii[6]

[1]Department of Electrical and Electronic Engineering, Kitami Institute of Technology, 165 Koen-cho, Kitami, Hokkaido 090-8507, Japan

[2]Department of Computer Science, Kitami Institute of Technology, 165 Koen-cho, Kitami, Hokkaido 090-8507, Japan

[3]Art, Science and Technology Center for Cooperative Research, Kyushu University, 6-1 Kasuga-koen, Kasuga, Fukuoka 816-8580, Japan

[4]Innovative Technology Research Center, Asahi Glass Co., Ltd., 1-1 Suehiro-cho, Tsurumi-ku, Yokohama, Kanagawa, 230-0045, Japan

[5]Innovative Technology Research Center, Asahi Glass Co., Ltd., 1150 Hazawa-cho, Kanagawa-ku, Yokohama, Kanagawa, 221-8755, Japan

[6]Research Institute for Electronic Science, Hokkaido University, Kita20, Nishi10, Kitaku, Sapporo, Hokkaido 001-0020, Japan

*d_sakai@mail.kitami-it.ac.jp

Relation between ion conductivity and space-selective deposition of SiO_2

Relationship between the ion conductivity of glass and the SiO_2 deposition during the corona discharge was investigated using two types of glasses with different ion conductivities. Figure S1 shows the experimental set up. The half area of the soda-lime glass of 25 mm × 25 mm × 1 mm was covered with a dehydrated synthetic fused silica glass plate of 12.5 mm × 25 mm × 0.5 mm. The ion conductivity of fused silica is negligible because of its extremely high purity. Figure S2 shows the top view of the specimen after the corona discharge treatment by an application of +6 kV to the needle electrode for 4 h at 200°C in air containing vaporized cyclic siloxane. The deposition of SiO_2 was confirmed only on the soda-lime glass surface which can be recognized by the formation of fringe pattern and the XPS analysis. On the other hand, there is no such deposition on the synthetic fused silica glass surface. Therefore, it is evident that the SiO_2 deposition during the corona discharge in the cyclic siloxane vapor depends on the ion conductivity of glass substrate.

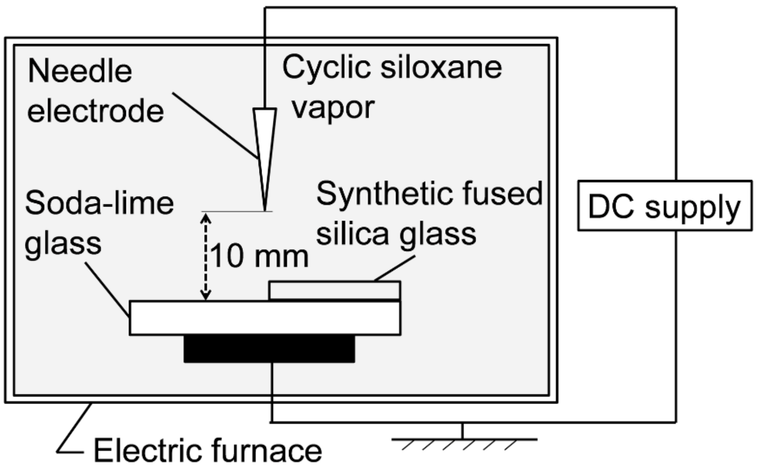

Figure S1 Experimental setup for corona discharge treatment of soda-lime glass in cyclic siloxane vapor. The half area of soda-lime glass was covered with synthetic fused silica glass.

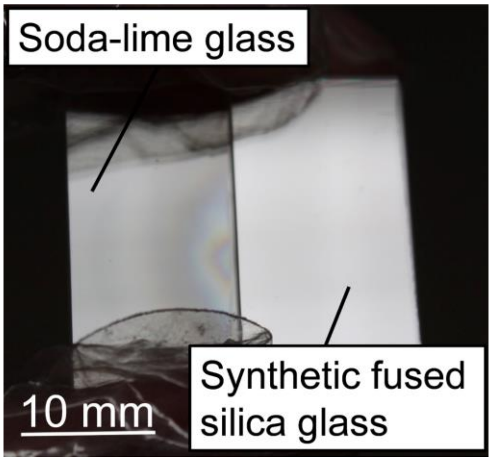

Figure S2 Photograph of the corona discharge treated soda-lime glass covered its half area with synthetic fused silica glass plate.
